# One more year and looking forward

**DOI:** 10.1007/s00167-020-06424-4

**Published:** 2021-01-22

**Authors:** Jon Karlsson, Volker Musahl, Michael T. Hirschmann

**Affiliations:** 1 Department of Orthopaedics, Sahlgrenska Academy, University of Gothenburg, Sahlgrenska University Hospital, 431 80 Mölndal, Gothenburg, Sweden; 2 Department of Orthopaedic Surgery, Center for Sports Medicine, UPMC Rooney, Pittsburgh Medical Center, 3200 S Water Street, Pittsburgh, PA 15203 USA; 3 Department of Orthopaedic Surgery and Traumatology, Kantonsspital Baselland (Bruderholz LiestalLaufen), 4101 Bruderholz, Switzerland

This has been a very strange year, related to the serious coronavirus infection that has affected all of us so heavily. Many changes, as elective surgery has been put on hold and many of us have worked part- or even full-time in the Corona health care. Obviously, the virus is a tough enemy and we are still not “rounding the corner”.
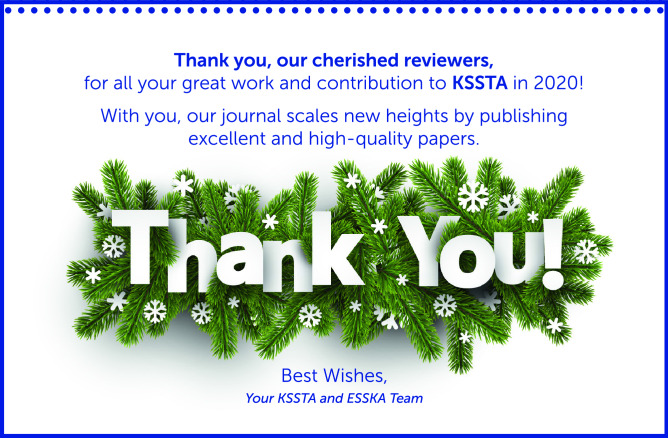


ESSKA, as a society, in collaboration with KSSTA, has published several useful guidelines on how to work and how to protect yourself during this predicament. We have during the year published several papers related to the virus crisis, these papers have been very well received. In some of them, we are looking forward: are we prepared to return to normal work when the quandary is over?

We have, in a joint effort with EKA, published many papers with relevant recommendations on how to maintain a safe work environment for hip and knee replacement surgeries and how we can safely return to work, when society slowly returns to normal.

There has been a major increase in submissions during the year. Usually, we receive approximately 100–140 manuscripts per month. But, during some months, we have received almost 250 submissions. We think that clinical researchers, who usually spend 3–4 days per week in the operation theatre, now have had more time to conduct research and write papers. Many of them are good and clinically valuable. For the first time, there will be over 2000 submissions this year.

This high volume inevitably means that the work load for all those involved in the journal on a daily basis has increased markedly. We would, therefore, like to thank all our reviewers who have had many more papers to review than earlier years. We would also like to thank the Associate Editors, the Editorial Board and last, but not least the Editorial Office, managed by Runeeta Rai and Simona Luparia, and the constant support offered by the ESSKA Executive Office, especially Zhanna Kovalchuck. Over 250 new submission per month, means that we have to read almost 8–10 new manuscripts every day, almost double of what we are used to. Thank you all again for your understanding and patience. We have been able to maintain our Impact Factor, which has exceeded 3.0 this year, in a manner similar to the last five years.

We have been successful on several projects, such as CME Credits for all reviews, a new feature and well liked by reviewers. We have considerably increased our teamwork with JEO, and now witness an increasing number of manuscripts that make their way to JEO for publication. We worked on the project “Women in ESSKA”, and we are proud that many of the young and bright women scientists in Europe are a part of our Editorial Board. We have also added several new Editorial Board Members and reviewers. We welcome and thank you all for the good work during 2020.

Finally, we would like to introduce our new Deputy Editor-in-Chief: Professor Olufemi Ayeni. He is the Division Head of Orthopaedic Surgery at McMaster University in Canada. He is also the Medical Director of the Hamilton Tiger Cats (Canadian Football League) and Forge FC (Canadian Premier League). He has published over 300 academic publications (peer-reviewed and book chapters) focused on sports medicine with a predominant focus on Femoroacetabular Impingement (FAI). His research focused on FAI has been funded by major institutions including the Canadian Orthopaedic Foundation, American Orthopaedic Society for Sports Medicine, Arthroscopy Association of North America and Canadian Institute for Health Research. Prof. Ayeni has presented widely across the globe on FAI, research methodology (including randomised controlled trials, systematic review and meta-analyses) and is the recipient of several prestigious awards. Recently, he was awarded the J. Edouard Samson Award (2020) for the best career Orthopaedic Research in Canada over a 5-year period. He has executive roles in several international organisations including ISAKOS and ISHA. Locally, his other administrative roles include Head of Orthopaedic Service at McMaster University Medical Centre, Fellowship Director for Sports Medicine and Arthroscopy and associate faculty member of the Department of Health Research Methods, Evidence and Impact (McMaster University). Over the years, Prof. Ayeni has served as a reviewer, Editorial Board member and Associate Editor for KSSTA, and we welcome him in his new role.
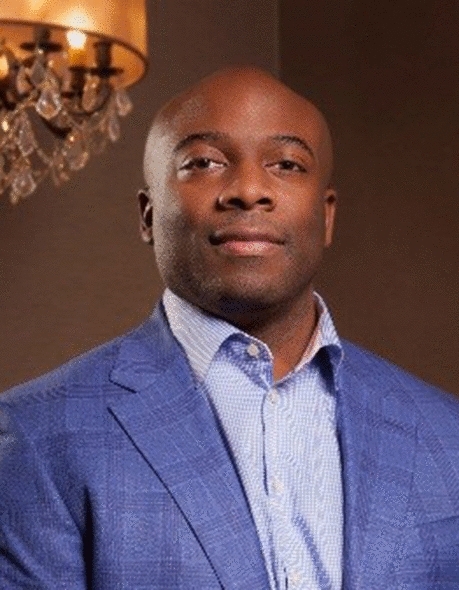


Thank you all for 2020. We look forward to 2021, when hopefully our lives will be back on track!

**Table:** 

First Name	Last Name
Giuseppe	Filardo
Antonio	Klasan
Daniel	Pérez Prieto
Kanto	Nagai
Jari	Dahmen
Theresa	Diermeier
Dominic	Mathis
Mo	Saffarini
Nobuo	Adachi
Baris	Kocaoglu
Florian	Imhoff
Henrik	Behrend
Riccardo	Compagnoni
Felix	Dyrna
Andreas	Imhoff
Thomas	Muellner
Geert	Pagenstert
Pietro	Randelli
Patrick	Sadoghi
Rene	Verdonk
Paulo	Araujo
Ulrich	Bosch
Kristín	Briem
Daniel	Guenther
Anna	Hirschmann
Yuichi	Hoshino
Albert	Lin
Stefan	Mogos
Takeshi	Muneta
Sufian	Ahmad
Philippe	Beaufils
Matthias	Brockmeyer
Björn	Drews
Trifon	Totlis
Philipp	Winkler
Enric	Castellet
Takanori	Iriuchishima
Gunnar	Knutsen
Robert	LaPrade
Khaled	Meknas
John	Nyland
Alberto	Ventura
Murat	Bozkurt
Thorben	Briese
Klaus	Draenert
Radu	Fleaca
Moin	Khan
Philippe	Massin
Tommaso	Roberti di Sarsina
Philip	Roessler
Benjamin	Rothrauff
Alex	Shearman
Andreas	Voss
Andrea	Achtnich
Mike	Carmont
Andreas	Gomoll
Alberto	Grassi
Michael	Hantes
Pedro	Hinarejos
Michael	Liebensteiner
Olaf	Lorbach
Miguel	Ruiz Iban
Romain	Seil
Michel P. J.	van den Bekerom
Marcio	Albers
Johannes	Beckmann
Paolo	Bulgheroni
Miki	Dalmau-Pastor
Riccardo	D'Ambrosi
Simon	Donell
Carl	Haasper
William	Hage
Giuseppe	Milano
Gilbert	Moatshe
Lukas	Moser
Axel	√ñhlin
Thomas	Tischer
Michele	Vasso
Patrick	Weber
Henrik	Aagaard
Elizabeth	Arendt
Knut	Beitzel
Hanna	Bjørnsson Hallgren
Martin	Clauss
Mahmut	Doral
Jon	Drogset
Karl	Eriksson
João	Espregueira-Mendes
Enrique	Gomez Barrena
Eric	Hamrin Senorski
Mustafa	Karahan
Nicola	Maffulli
Giulio Maria	Marcheggiani Muccioli
Wolfgang	Nebelung
Matthieu	Ollivier
Gunter	Spahn
Philippe	Tscholl
Carola	van Eck
Eva	Zeisig
Prashanth	Anand
Tommaso	Bonanzinga
Nicolaas	Budhiparama
Chong Bum	Chang
Johannes	Glasbrenner
Philipp	Heuberer
Xiaobin	Huang
Eoghan	Hurley
Michael	Iosifidis
Tomoya	Iseki
Jae-Heon	Jeong
Jefferey	Kay
Shwan	Khoschnau
Hideyuki	Koga
Keith	Lawhorn
Parth	Lodhia
Jeffrey	Macalena
Mahir	Mahirogullari
Martin	Pietsch
Elliot	Sappey-Marinier
Bruno	Violante
Harald	Widhalm
Chaochao	Zhou
Christoph	Becher
Annelie	Brorsson
Eivind	Inderhaug
Christoph	Kittl
Andreas	Persson
Claudio	Rosso
Gonzalo	Samitier
Mattias	Ahldén
Akram	Aldawoudy
Cecile	Batailler
Rogerio	Carvalho
Etienne	Cavaignac
Simone	Cerciello
Cheng-Kung	Cheng
Mark	Clatworthy
Vincenzo	Condello
Davide	Cucchi
John	Gliatis
Alfred	Hochrein
Jonathan	Hughes
Mark	Hutchinson
Edna	Iordache
Jakub	Kautzner
Matthias	Krause
Ricardo	Larrainzar-Garijo
Geert	Meermans
Muzammil	Memon
Céline	Moret
Thierry	Pauyo
Christian	Peez
Nicolas	Pujol
Maristella	Saccomanno
Andrew	Sheean
Seth	Sherman
Sebastian	Siebenlist
Inge	Skråmm
Pietro	Spennacchio
Katja	Tecklenburg
Marc	Tompkins
Manuel	Vieira da Silva
Alastair	Younger
Mike	Baums
Onur	Bilge
David	Dejour
Francesco	Giron
Christophe	Hulet
Mislav	Jelic
Oliver	Kessler
Sebastien	Lustig
Katarina	Nilsson-Helander
Thilo	Patzer
Christopher	Pearce
Luigi	Pederzini
Eleonor	Svantesson
Jeffrey	Abrams
Michael	Alaia
Jack	Andrish
Rene	Attal
Jesper	Augustsson
Adad	Baranto
Rudi	Bitsch
Berte	Boe
Robert	Brophy
Fabio	Conteduca
Iswadi	Damasena
Darren	de SA
Guillaume	Demey
Max	Ettinger
Mario	Ferretti
Pablo	Gelber
Silvan	Hess
Stephen	Howell
John	Kennedy
Jason	Koh
Elizaveta	Kon
Ioannis	Kostogiannis
Artur	Kroll
Dae-Hee	Lee
Peter	Leeuw
Pisit	Lertwanich
Guoan	Li
Nina	Magnitskaya
Stefan	Marlovits
Frank	Martetschläger
Bogdan	Matache
Craig	Mauro
Julian	Mehl
Juan	Monllau
Norimasa	Nakamura
Patrick	Orth
Charles	Riviere
Nick	Smith
Christian	Stärke
Robert	Steensen
Simon	Thompson
William	Walsh
Lukas	Willinger
Frantzeska	Zampeli
Oskar	Zupanc
Peter	Balcarek
Thomas	Bauer
Lieven	De Wilde
Joerg	Jerosch
Jörg	Lützner
Fabrizio	Margheritini
Hermann Otto	Mayr
Anders	Stalman
Pierluigi	Antinolfi
Paolo	Arrigoni
Bj√∂rn	Barenius
Etienne	Belzile
Massimo	Berruto
Daniel	Berthold
Tahsin	Beyzadeoglu
Lars	Blond
Reinoud W.	Brouwer
Jeremy	Burnham
Filippo	Calanna
Carlo	Camathias
Georgios	Chalatsis
Ricardo	Cuellar
Masataka	Deie
Jörg	Dickschas
Fredrik	Einarsson
Guri	Ekås
Mehmet	Erdil
Matthias	Feucht
Gianluca	Gallinari
Tobias	Gensior
Thomas	Gill
Mark	Glazebrook
Seung-Beom	Han
Joerg	Harrer
Petra	Heesterbeek
Jean-Yves	Jenny
Defne	Kaya
Vikas	Khanduja
Koichi	Kobayashi
Ladislav	Kovacic
Werner	Krutsch
Bryson	Lesniak
Sven	Lichtenberg
Bent	Lund
Mari	Lundberg
Inigo	Martinez
Tomoyuki	Matsumoto
Sean	Meredith
Duncan	Meuffels
Håvard	Moksnes
Lukas	Muench
Sebastian	Müller
Philipp	Niemeyer
Michael	O'Malley
Evangelos	Pappas
Yun	Peng
Devin	Peterson
Chadwick	Prodromos
Luca	Pulici
Filippo	Randelli
Mikel	Reilingh
Justin	Roe
Armin	Runer
Octav	Russu
Massimiliano	Salvi
Mikael	Sansone
Alfredo	Schiavone Panni
Reinhard	Schuh
Konsei	Shino
Paweł	Skowronek
Christoph	Theil
Soshi	Uchida
Christiaan	van Bergen
Pim	van Klij
Egbert	Veen
Thomas	Wood
Tobias	Wörner
Diederick	Wouters
Nestor	Zurita
Antonia	Chen
Laura	De Girolamo
Turgay	Efe
Alan	Getgood
Martin	Lind
Jacques	Menetrey
Caroline	Mouton
Kristian	Samuelsson
Kazunori	Yasuda
Franck	Accadbled
Paul	Ackermann
Ralph	Akoto
Khalid	Alkhelaifi
Tjarco	Alta
Ana Catarina	Ângelo
Daisuke	Araki
Raul	Barco
Kristoffer	Barfod
Himanshu	Bhayana
Kerem	Bilsel
Gerrit	Bode
Friedrich	Boettner
Michel	Bonnin
Georg	Brandl
Martijn	Brinkman
Mats	Brittberg
Charles	Brown
Ofir	Chechik
Hakan	Cift
Thomas	Clanton
Hywel	Davies
Thomas	De Bo
Ricardo	de Casas
Koen	Defoort
Neel	Desai
Brian	Devitt
Pieter	D'Hooghe
Marcin	Domzalski
Matej	Drobnic
Lars	Ejerhed
Younes	El moudni
Selim	Ergün
Alejandro	Espejo-Reina
Simon A.	Euler
Filippo	Familiari
Peter	Fauno
Christian	Fink
Mark	Fontana
Amy	Garner
Ron	Gilat
Antonia	Gkotsi
Erin	Gordey
Nicolas	Graveleau
Stefan	Grote
Martin	Hägglund
Sherwan	Hamawandi
Horia	Haragus
Kazuhisa	Hatayama
Philipp	Henle
MaCalus	Hogan
Christian	Hoser
Kaywan	Izadpanah
Vincenzo	Izzo
Jayadeep	Jayachandran
Nayana	Joshi-Jubert
Peter	Kälebo
Mai	Katakura
Jan Christoph	Katthagen
Iza√§k	Kodde
Georgios	Komnos
Matthias	Königshausen
Rik	Kundra
Lior	Laver
Nicolas	Lefevre
Maria Joao	Leite
Ofer	Levy
Denny	Lie
Emilio	Lopez-Vidriero
Nicola	Lopomo
Angelina	Lukaszenko
Robert	Magnussen
Konstantinos	Makridis
Philipp	Michel
Andreas	Mueller
Bartosz	Musielak
Alex	Nedopil
Geoffroy	Nourissat
Marko	Ostojiƒá
Christian	Owesen
Nikolaos	Paschos
Rahul	Patel
Giuseppe	Peretti
Frank	Petrigliano
Jonas	Pogorzelski
Andrew	Porteous
Per-Henrik	Randsborg
Vasileios	Raoulis
Bjoern	Rath
Quinten	Rikken
Leho	Rips
Patrick	Robinson
Scott	Rodeo
Juan	Rodríguez-Roiz
Jan Harald	Røtterud
Adnan	Saithna
Björn	Salomonsson
Oliver	Schindler
Benedikt	Schliemann
Kristian	Schneider
Feisal	Shah
Kazunori	Shimomura
Thorkell	Snaebjörnsson
Francesc	Soler
Eirik	Solheim
Bertrand	Sonnery-Cottet
Thomas	Stein
Amelie	Stoehr
Tamer	Sweed
Masato	Takao
Fritz	Thorey
Cecile	Toanen
Roy	Tranberg
Elias	Tsepis
Sebastiaan	van de Groes
Anne	van der Made
Derek	van Deurzen
Nicolien	Van Giffen
Nicky	van Melick
Joao	Vide
Marco	Viganò
Jonas	Werner
Raymond	Yeak
Shinichi	Yoshiya
Giacomo	Zanon
Urszula	Zdanowicz
Jennifer	Zellers
Eline	Zwitser

